# Dual-mode nanoprobe strategy integrating ultrasound and near-infrared light for targeted and synergistic arterial thrombolysis

**DOI:** 10.1186/s12951-024-02562-w

**Published:** 2024-06-03

**Authors:** Zhiwen Wang, Nan Jiang, Zhixin Jiang, Hao Wang, Yuxin Guo, Fanglu Zhong, Bin Gui, Yueying Chen, Qing Deng, Qing Zhou, Bo Hu

**Affiliations:** 1 Echo Lab, Department of Ultrasound Imaging, Renmin Hospital of Wuhan University, 238# Jiefang Road, Wuhan, 430060 Hubei People’s Republic of China; 2 Hubei Key Laboratory of Cardiology, Wuhan, 430060 People’s Republic of China; 3 Cardiovascular Research Institute, Wuhan University, Wuhan, 430060 People’s Republic of China

**Keywords:** Sonothrombolysis, Photothermal thrombolysis, Nanoprobe, Ultrasound, Near-Infrared Light

## Abstract

Efficient thrombolysis in time is crucial for prognostic improvement of patients with acute arterial thromboembolic disease, while limitations and complications still exist in conventional thrombolytic treatment methods. Herein, our study sought to investigate a novel dual-mode strategy that integrated ultrasound (US) and near-infrared light (NIR) with establishment of hollow mesoporous silica nanoprobe (HMSN) which contains Arginine-glycine-aspartate (RGD) peptide (thrombus targeting), perfluoropentane (PFP) (thrombolysis with phase-change and stable cavitation) and indocyanine green (ICG) (thrombolysis with photothermal conversion). HMSN is used as the carrier, the surface is coupled with targeted RGD to achieve high targeting and permeability of thrombus, PFP and ICG are loaded to achieve the collaborative diagnosis and treatment of thrombus by US and NIR, so as to provide a new strategy for the integration of diagnosis and treatment of arterial thrombus. From the in vitro and in vivo evaluation, RGD/ICG/PFP@HMSN can aggregate and penetrate at the site of thrombus, and finally establish the dual-mode directional development and thrombolytic treatment under the synergistic effect of US and NIR, providing strong technical support for the accurate diagnosis and treatment of arterial thrombosis.

**Graphical Abstract:**

**Figure:**
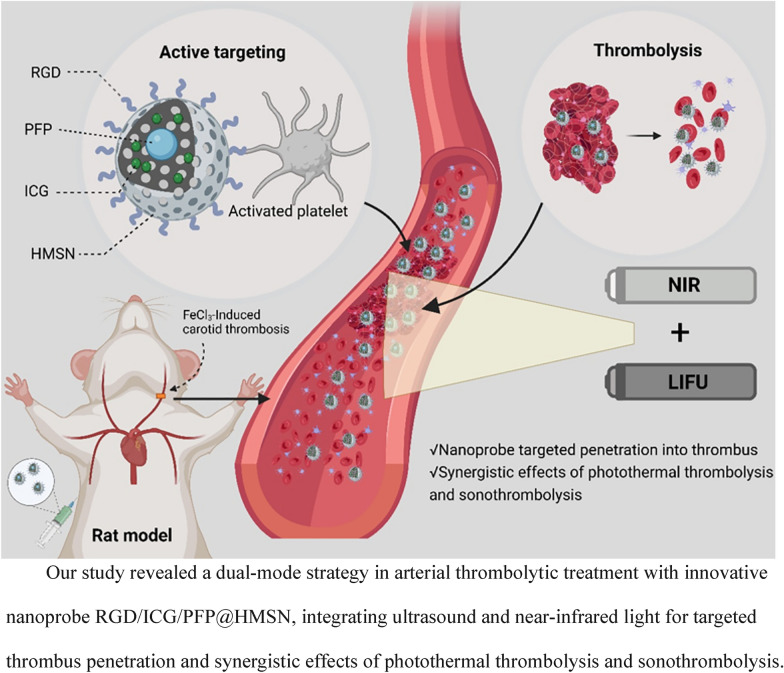

## Introduction

Thrombosis can lead to blood vessel obstruction, resulting in a significant decrease in blood flow and posing a threat to vital organ function and the patient's life [1, 2]. Conventional treatments for thrombus elimination in clinical practice include medication, intervention [3], or surgery. However, these methods still face challenges such as uncertain thromboembolism diagnosis, limited application range, and potential complications [4–7]. For instance, thrombolytic drug therapy may be limited by the treatment window and risks of systemic bleeding [8, 9], while vascular intervention and surgery may yield unsatisfactory results and invasive complications, significantly affecting patient prognosis.

In response to these limitations, researchers have explored more convenient and non-invasive methods for thrombolysis, such as sonothrombolysis (ultrasound-mediated thrombolysis) and photothermal thrombolysis. Sonothrombolysis leverages acoustic particles like microbubbles to disrupt the thrombus fiber network structure through mechanical mechanisms like cavitation and resonance effects [10–12]. On the other hand, photothermal thrombolysis raises the temperature to destroy the non-covalent bond of fibrin in the thrombus, enlarging the tissue gap of the thrombus and improving drug permeability, thereby enhancing the antithrombotic effect [13, 14]. While photothermal therapy alone might not be sufficient to eliminate the thrombus entirely [14], the development of a nanoprobe targeting thrombus, in combination with sonothrombolysis, could offer a novel strategy to overcome the limitations of conventional treatment methods.

Photosensitizers utilized in photothermal therapy serve a dual purpose, not only enabling photothermal thrombolysis but also facilitating near-infrared thrombus imaging. Among these photosensitizers, indocyanine green (ICG), a near-infrared fluorescent dye, stands out due to its excellent safety profile, short half-life, and efficient photothermal conversion capabilities [15, 16]. Hence, ICG can play a therapeutic role in thrombolysis while providing high-quality optical imaging of arteries. To enhance sonothrombolysis, we have opted for nanodroplets as phase transition agents, which can penetrate the thrombus and unleash their effects when responsive to US [17]. Perfluoropentane (PFP), a nanoscale liquid fluorocarbon, exhibits favorable biosafety and phase transition properties [18, 19]. Upon low-intensity focused ultrasound (LIFU) excitation, PFP transitions from a liquid to a gas state, amplifying the ultrasound signal and inducing cavitation and other thrombolysis effects [20]. Additionally, PFP undergoes thermal-induced phase transition under near-infrared (NIR) irradiation, facilitating the delivery of microbubbles to enhance the acoustic thrombolysis effect and improve the thrombolysis rate [21]. Our study has chosen hollow mesoporous silica nanoparticles (HMSN) as the carrier due to the modifiable properties and excellent biocompatibility [22, 23]. These nanoparticles are equipped with ICG and PFP, enabling the integration of dual-mode diagnosis and treatment under US and NIR irradiation. This innovative approach aims to optimize thrombolysis efficiency and promote targeted therapy for improved clinical outcomes.

To facilitate the precise targeting of the nanoprobe to the thrombus site within the circulation, we incorporated an arginine glycine aspartic (RGD) sequence into the system. This RGD sequence possesses the capability to bind specifically to the platelet glycoprotein GPIIb/IIIa receptor present in the thrombus [10, 24, 25]. In our therapeutic strategy, the nanoprobe is devoid of thrombolytic agents; instead, it is equipped with thrombus-targeting RGD sequences. The selective thrombolytic action is facilitated by cavitation and photothermal effects induced by the nanoprobe under the guidance of US/NIR stimulation. By introducing the RGD sequences, we can effectively circumvent the high risk of off-target bleeding associated with conventional thrombolytic therapies. The incorporation of RGD enables the nanoprobe to home in on and penetrate the thrombus, leveraging both sonothrombolysis and photothermal thrombolysis to promote thrombus lysis at a deeper level. This creates a positive cycle of "lysis infiltration lysis," effectively maximizing the thrombolytic efficacy of the treatment.

**Fig. 1:**
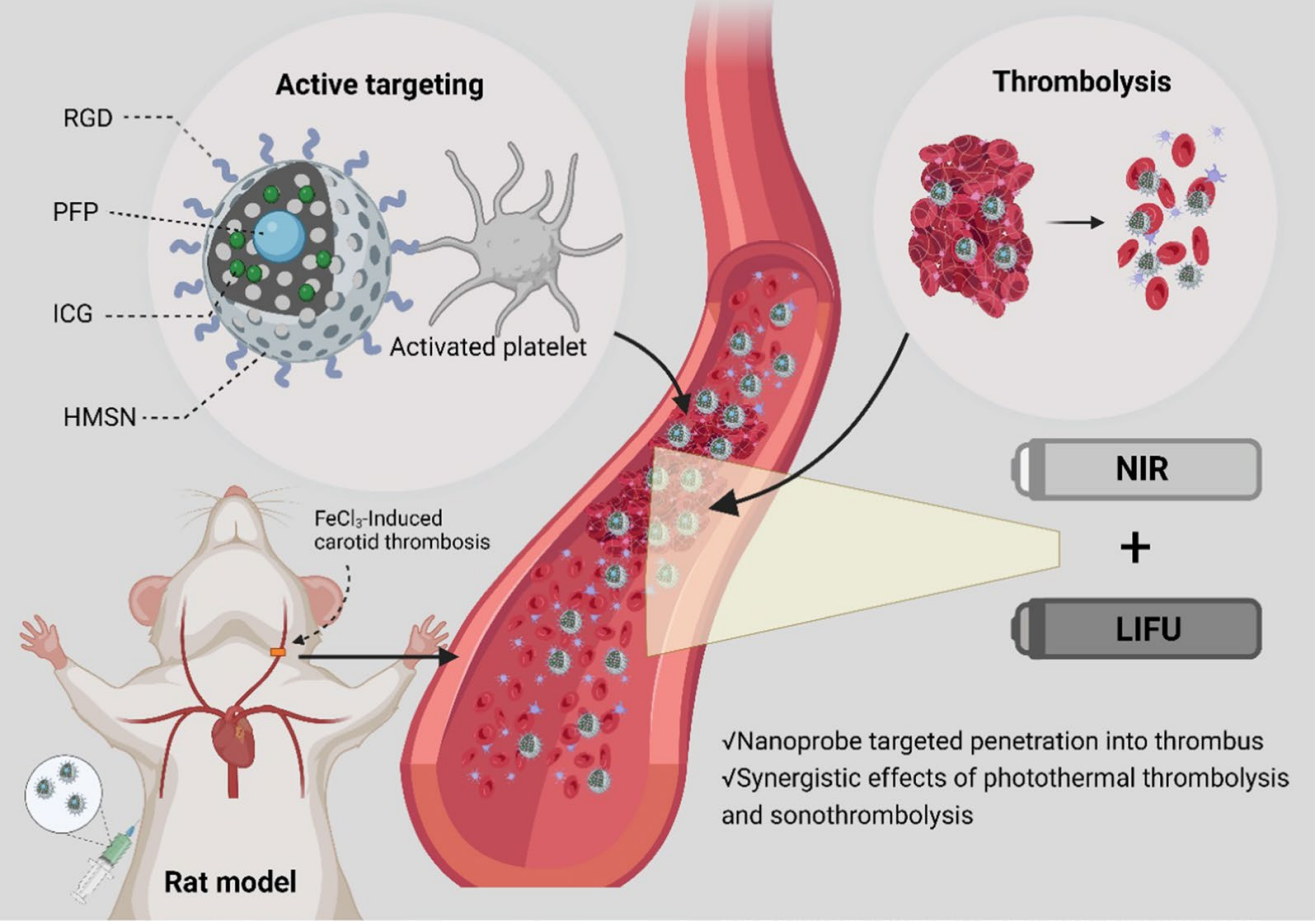
Schematic diagram of RGD/ICG/PFP@HMSN targeting thrombus under the synergistic effect of ultrasound and near-infrared light irradiation

In this study, we present a novel integrated dual-mode imaging nanoprobe designed for targeted diagnosis and treatment, named RGD/ICG/PFP@HMSN. The schematic diagram is summarized in Fig. 1. The nanoprobe utilizes HMSN as the carrier, with targeted RGD on its surface to enhance specific binding and permeability to the thrombus site. Furthermore, it is loaded with PFP and ICG, enabling collaborative diagnosis and treatment of the thrombus using both US and NIR imaging modalities. Through this approach, we aim to explore the potential of US and NIR dual-mode imaging and the synergistic effect of thrombolysis. Our study provides a promising new strategy for integrating the diagnosis and treatment of arterial thrombus, advancing the field of thrombus management.

## Results and discussion

### Characteristics of RGD/ICG/PFP@HMSN

**Fig. 2:**
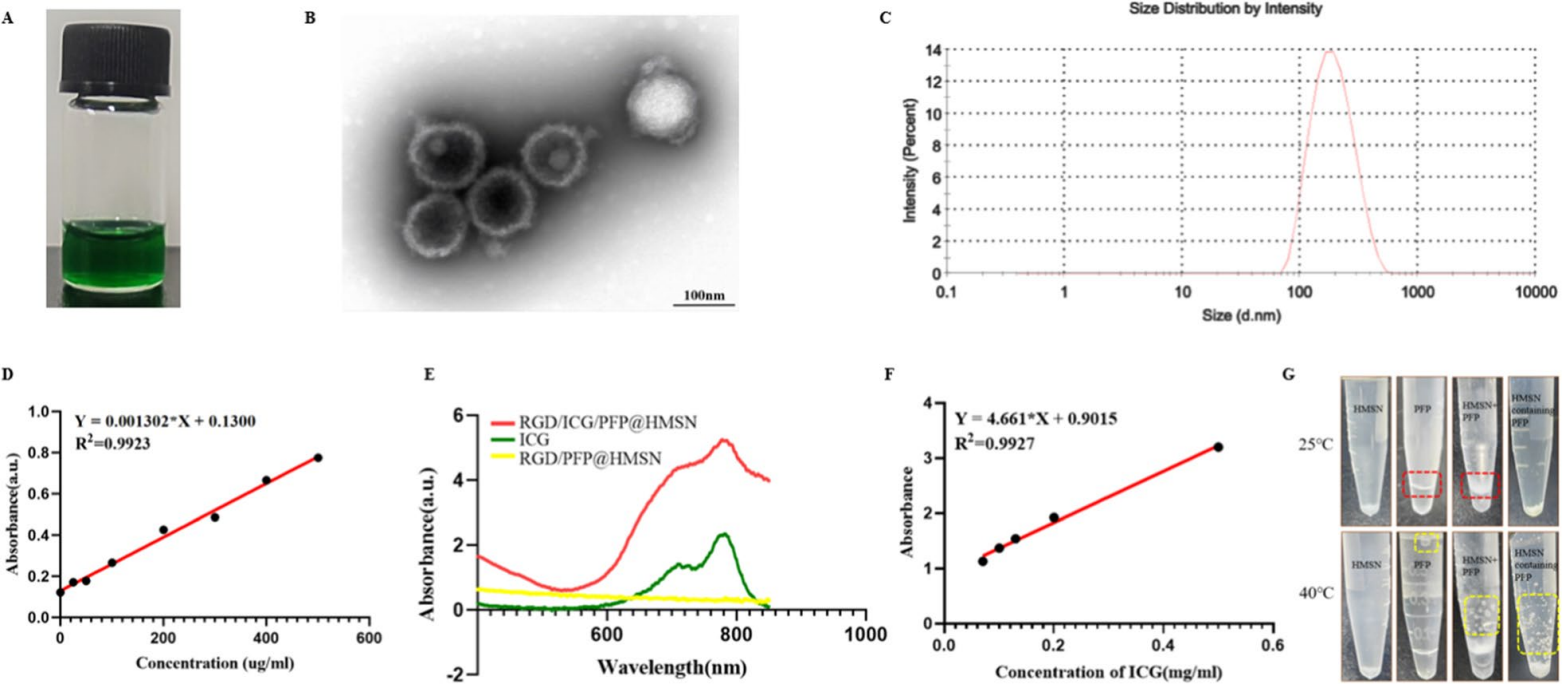
Characterization of RGD/ICG/PFP@HMSN nanoprobe. **A** Green suspension appearance of the nanoprobe. **B** TEM imaging of the nanoprobe. **C** Particle size analysis of the nanoprobe. **D** Standard curve for BCA analysis. **E** UV visible near-infrared spectroscopy results. **F** Standard curve for ICG loading. **G** Comparative study involving HMSN, PFP, the physical mixture of HMSN and PFP (HMSN + PFP), HMSN containing PFP in pure water at 25 °C and 40 °C. The red dotted box indicates water-PFP interface and yellow box represents the formation of bubbles

The synthesis of RGD/ICG/PFP@HMSN was primarily achieved through ultrasonic vibration and carbodiimide method. This resulted in the formation of a green suspension (Fig. 2A). TEM imaging revealed that RGD/ICG/PFP@HMSN exhibited a spherical shape, demonstrating the observation of the process of PFP phase transition within HMSN at different stages: initially absent (perhaps because PFP had not yet undergone phase transition or had already undergone phase transition into gas escaped from the pores of HMSN), then PFP transformed into a small sphere and occupying the entire inner space of HMSN (Fig. 2B). The hydrated particle size of RGD/ICG/PFP@HMSN was (181.1 ± 5.35) nm (Fig. 2C), and the nanoscale size facilitated the nanoprobe’s deep penetration into the thrombus, enhancing its effectiveness.

To confirm the successful coupling of RGD, the BCA method was employed due to its capability to detect proteins and perform quantitative analysis. The coupling rate of RGD was calculated to be 95.78% ± 0.21% (Fig. 2D), laying a solid foundation for subsequent thrombus-targeting experiments. UV visible near-infrared spectroscopy was used to display characteristic ICG peaks on the nanoprobe, thus confirming the successful loading of ICG (Fig. 2E). The standard curve for ICG was established as y = 4.661x + 0.9015, and based on the standard curve, the encapsulation efficiency of ICG was determined to be 97.30% ± 0.05% (Fig. 2F). The high encapsulation efficiency of ICG was advantageous for NIR imaging and photothermal therapy using the nanoprobes.

Our experiments have confirmed the successful loading of PFP into the nanoprobe RGD/ICG/PFP@HMSN from various perspectives. These findings demonstrate the nanoprobe's efficient PFP loading and its phase transition properties. In the PFP loading experiment, no changes were observed in the HMSN group before and after incubation. In contrast, PFP, which is insoluble in water, displayed a distinct boundary in the water. Following incubation, a phase transition occurred, and bubbles formed in the water. Loading PFP into HMSN caused the boundary to disappear, yet bubbles still appeared in the water after incubation, confirming the phase transition and successful PFP loading in HMSN (Fig. 2G).

### Dual-mode imaging properties of the RGD/ICG/PFP@HMSN in vitro

**Fig. 3:**
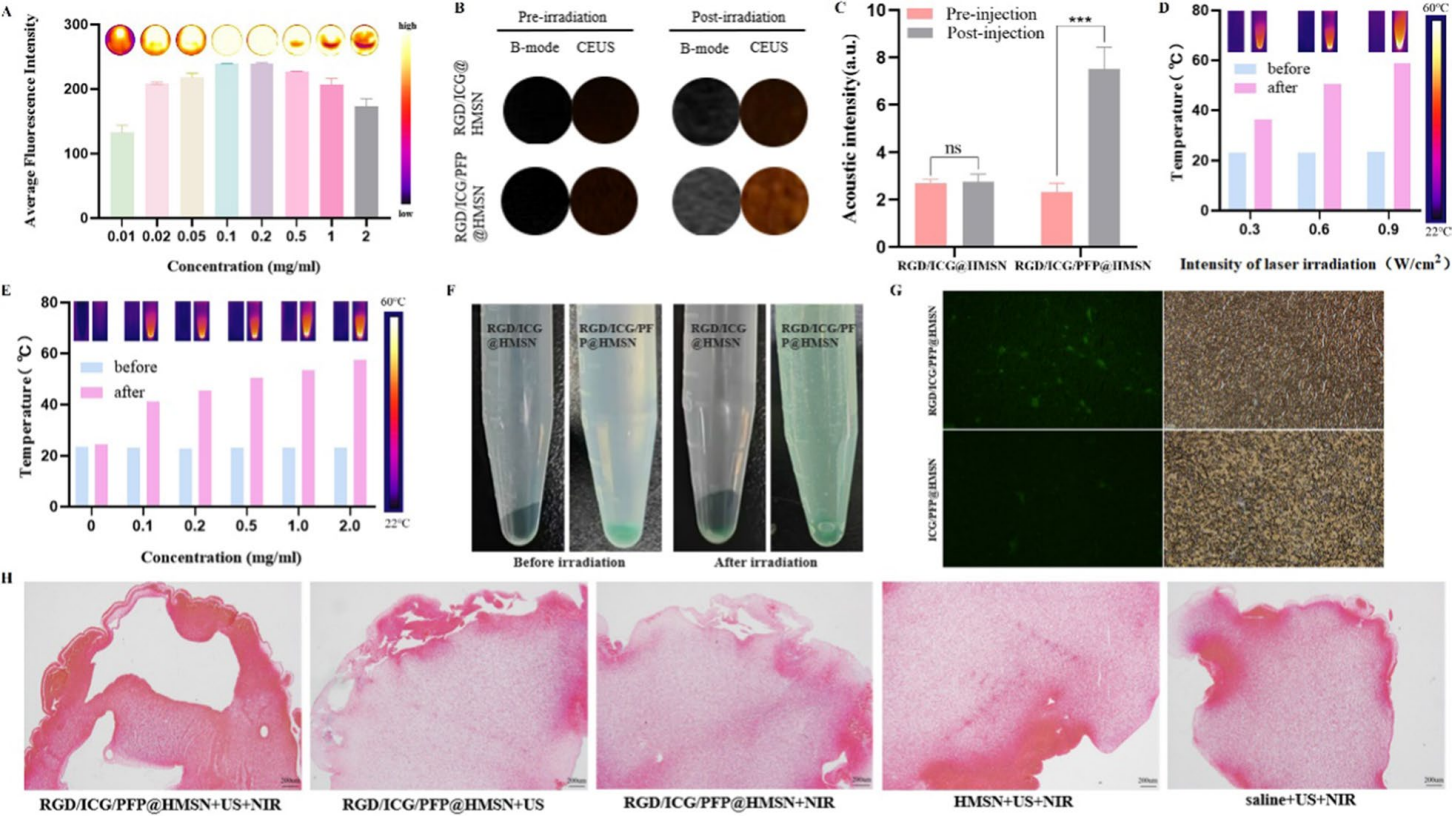
In Vitro Assessment of RGD/ICG/PFP@HMSN: Dual-Mode Imaging, Photothermal Conversion, Clot Binding, and Thrombolysis. **A** In vitro NIR imaging of the nanoprobe. **B** In vitro US imaging of the nanoprobe. **C** Quantitative analysis of contrast-enhanced acoustic signal intensity in vitro. **D** Photothermal conversion efficiency of the nanoprobe at different laser intensities. **E** Photothermal conversion efficiency of the nanoprobe at different concentrations. **F** Phase transition of RGD/ICG/PFP@HMSN and RGD/ICG@HMSN before and after laser irradiation. **G** Assessment of RGD/ICG/PFP@HMSN and ICG/PFP@HMSN binding to clots (× 200). **H** Thrombolytic pathological sections of each group

In the context of NIR imaging, the NIR live imaging system unveiled a trend where the near-infrared fluorescence signal gradually intensified and then decreased as the concentration of RGD/ICG/PFP@HMSN increased (Fig. 3A). This behavior can be attributed to the self-quenching of imaging signal due to the elevated ICG concentration [26], which holds significance as a reference for selecting the appropriate nanoprobe concentration for in vivo applications.

Concerning US imaging, both RGD/ICG@HMSN and RGD/ICG/PFP@HMSN initially exhibited low signal intensity in both grayscale and contrast modes before being subjected to low-intensity focused ultrasound irradiation. Following irradiation, RGD/ICG@HMSN continued to display a low signal in both grayscale and contrast modes, while the acoustic signal of RGD/ICG/PFP@HMSN experienced a significant intensification (Fig. 3B, C). This clearly demonstrated that RGD/ICG/PFP@HMSN nanoprobe’s excellent phase transition ability from liquid to gas state under US irradiation.

The results obtained from NIR and US imaging underscore the outstanding dual-mode imaging capabilities of the nanoprobe, establishing a solid foundation for subsequent in vivo experiments.

### Photothermal conversion efficiency and thermotropic phase transition in vitro

Following irradiation with an 808 nm laser of the same intensity (0.6 W), different concentrations of RGD/ICG/PFP@HMSN displayed increasing temperatures as the nanoprobe concentration gradually rose. Consequently, the heat map became brighter with these corresponding temperature increases. In contrast, the control group (pure water) showed no significant temperature elevation (Fig. 3D).

Subsequently, when nanoprobes of the same concentration (0.5 mg/mL) was exposed to varying intensities of the 808 nm laser, a direct correlation was observed: higher laser intensities led to greater temperature elevations in the nanoprobe, accompanied by brighter heat maps (Fig. 3E). These observations underscore the exceptional photothermal conversion capability of the nanoprobe, which exhibits concentration and laser intensity dependence. Consequently, precise selection of nanoprobe concentration and laser intensity is essential for optimizing thrombolysis efficiency while ensuring safety.

Before and after laser irradiation, the ICG/PFP@HMSN group displayed no observable changes, while the RGD/ICG/PFP@HMSN group exhibited the formation of bubbles post-irradiation, indicating a phase transition in PFP. During this phase transition, the nanoprobe at the lower portion was agitated and elevated due to the bubble generation, resulting in a greener appearance of the solution (Fig. 3F). This phenomenon demonstrated the nanoprobe's capacity to induce thermal phase transition, further enhancing its efficacy for subsequent photothermal thrombolysis.

### In Vitro binding capability and thrombolysis potential of RGD/ICG/PFP@HMSN

Cryosectioning results revealed that even after thorough rinsing, the arterial thrombus co-incubated with RGD/ICG/PFP@HMSN displayed a vibrant green fluorescence signal, surpassing that of the ICG/PFP@HMSN group (Fig. 3G). This observation highlighted the effective targeting ability of RGD/ICG/PFP@HMSN towards arterial thrombus.

In the thrombolysis experiment, RGD/ICG/PFP@HMSN + US + NIR group demonstrated a thrombolysis rate of 48.17% ± 0.89%, notably higher than that of RGD/ICG/PFP@HMSN + US group (38.54% ± 1.27%), RGD/ICG/PFP@HMSN + NIR group (33.93% ± 1.48%), HMSN + US + NIR group (20.84% ± 3.21%), and saline + US + NIR group (18.60% ± 1.93%) (P < 0.01, all) (Table 1). Pathological sections depicted multiple larger voids on the thrombus surface in RGD/ICG/PFP@HMSN + US + NIR group compared to other groups (Fig. 3H), indicative of the superior thrombolytic efficacy of the nanoprobe under the influence of both US and NIR irradiation. This illustrated that the synergistic effect of US and NIR can maximize the thrombolytic effect compared to US or NIR therapy alone. The fibrous protein framework within the thrombus is significantly disrupted, causing an increase in the spacing between fibrin chains within the thrombus. This enhances the "porosity" of the thrombus, facilitating its breakdown and leading to the rapid disintegration into tiny particles, thereby promoting the process of thrombus dissolution in a very short period of time. Thus, the synergistic combination of US and NIR has layed the foundation for subsequent in vivo thrombolytic therapy.

**Table 1:** Thrombolytic results among groups (x̅ ± s)

Group	Weight of blood clots before irradiation (mg)	Weight of blood clots after irradiation (mg)	Weight loss (mg)	Thrombolysis rate
RGD/ICG/PFP@HMSN + US + NIR	113.6 ± 2.11	58.9 ± 2.81	54.7 ± 1.64	(48.17 ± 0.89)%
RGD/ICG/PFP@HMSN + US	113.4 ± 3.45	69.73 ± 3.58	43.67 ± 0.12	(38.54 ± 1.27)%^a^
RGD/ICG/PFP@HMSN + NIR	111.73 ± 6.26	73.77 ± 3.54	37.97 ± 3.35	(33.93 ± 1.48)%^a^
HMSN + US + NIR	108.6 ± 4.18	85.87 ± 2.80	22.73 ± 4.06	(20.84 ± 3.21)%^a,b,c^
Saline + US + NIR	107.97 ± 4.61	87.8 ± 2.48	20.17 ± 2.85	(18.60 ± 1.93)%^a,b,c^

^a^Compared with Group RGD/ICG/PFP@HMSN + US + NIR, *P* < 0.01

^b^Compared with Group RGD/ICG/PFP@HMSN + US, *P* < 0.01

^c^Compared with Group RGD/ICG/PFP@HMSN + NIR, *P* < 0.01

### Dual-mode imaging for successful thrombus targeting in vivo

**Fig. 4:**
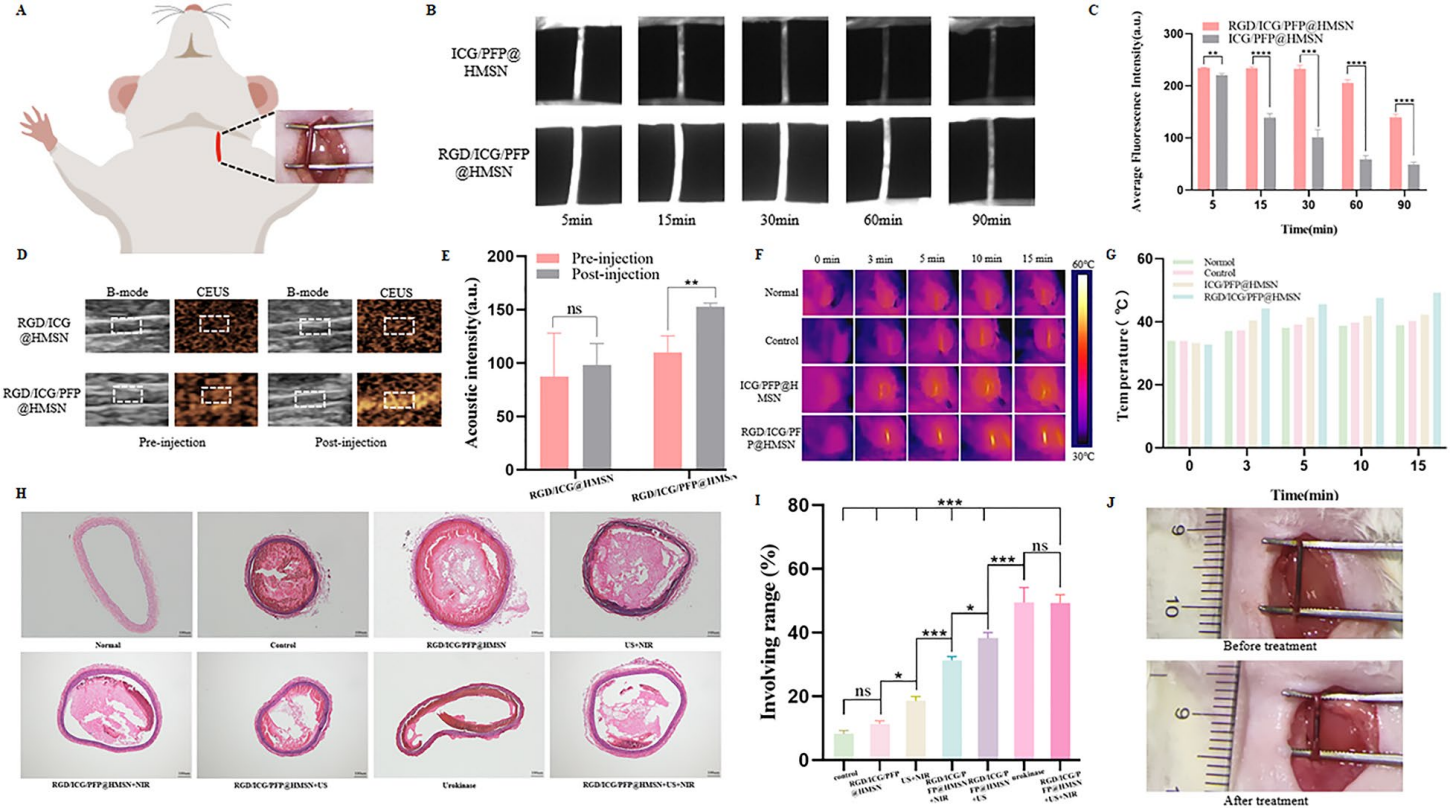
Dual-mode imaging, Photothermal conversion efficiency and Site-specific thrombolysis in vivo. **A** Schematic diagram of left carotid artery in rats. **B** Fluorescence images of left carotid artery thrombotic vessels in rats at different time points after the injection of ICG/PFP@HMSN and RGD/ICG/PFP@HMSN. **C** Fluorescence intensity of left carotid artery thrombotic vessels in rats at different time points after injection of ICG/PFP@HMSN and RGD/ICG/PFP@HMSN. n = 3, ***p < 0.001. **D** B-mode and CEUS of left carotid artery thrombotic vessels in rats after injection of ICG/PFP@HMSN and RGD/ICG/PFP@HMSN. **E** Quantitative analysis of contrast-enhanced acoustic signal intensity in vivo. **F** Corresponding thermal images of the thrombus site after NIR irradiation. **G** Temperature changes of left carotid artery thrombotic vessels in rats after NIR irradiation (0.6 W/cm^2^). **H** HE staining of the left carotid artery after treatment. n = 3. **I** Extent of thrombolysis effects coverage. n = 3, *p < 0.05, ***p < 0.001. **J** Photographs of the carotid artery embolization in rats before or after treatment

In the context of NIR imaging, our study with SD rats yielded compelling results. Thirty minutes following the injection of RGD/ICG/PFP@HMSN, a strong and sustained fluorescence intensity persisted at the sites of obstructed blood vessels, while the fluorescence intensity after the injection of ICG/PFP@HMSN at the same time point remained notably weak (Fig. 4A–C). This underscores the superior thrombus-targeting capability of RGD/ICG/PFP@HMSN, indicating its sustained affinity for thrombus. Additionally, the changes in fluorescence intensity within obstructed blood vessels over the 90-min post-injection period (Fig. 4B) provide valuable insights for determining optimal treatment durations in vivo. Also, we found that NIR imaging exhibits superior imaging capabilities, particularly in terms of excellent spatial resolution for imaging factors such as microvascular perfusion and vascular boundaries within small blood vessels.

Shifting our focus to US imaging, the results were equally promising. In the RGD/ICG/PFP@HMSN group, there was a remarkable increase in the acoustic signal intensity within the thrombus region (Fig. 4D, E). In contrast, the echo signal in the ICG/PFP@HMSN group remained subdued within the same region. This phenomenon signifies the nanoprobe’s capacity to undergo a liquid–gas phase transition upon US irradiation, thereby amplifying the US signal. Notably, this effect also plays a pivotal role in harnessing the cavitation phenomenon for potentiated thrombolytic therapy.

### Dual-mode treatment for efficient and synergistic thrombolysis in vivo

RGD/ICG/PFP@HMSN exhibited remarkable photothermal conversion ability in comparison to the other groups in vivo. Under NIR irradiation, the temperature of the left carotid artery in rats increased by approximately 5.0 °C and 6.4 °C in the normal group (the untreated rat carotid arteries as the normal group) and control group (the rat left carotid artery thrombosis model as the control group), respectively. In contrast, it increased by approximately 9.1 °C and 16.6 °C in the ICG/PFP@HMSN group (the rat left carotid artery thrombosis model injected with ICG/PFP@HMSN) and RGD/ICG/PFP@HMSN group (the rat left carotid artery thrombosis model injected with RGD/ICG/PFP@HMSN). After NIR irradiation, the temperatures of each group (normal group, control group, ICG/PFP@HMSN group, RGD/ICG/PFP@HMSN group) reached approximately 39.0 °C, 40.3 °C, 42.3 °C and 49.4 °C (Fig. 4F, G). This strong photothermal conversion ability of RGD/ICG/PFP@HMSN laid the foundation for its photothermal thrombolysis effect.

The investigation into thrombolysis effectiveness revealed that the thrombolysis rates of each group (control group, RGD/ICG/PFP@HMSN group, US + NIR group, RGD/ICG/PFP@HMSN + NIR group, RGD/ICG/PFP@HMSN + US group, urokinase group, and RGD/ICG/PFP@HMSN + US + NIR group) were 8.21%, 11.36%, 18.54%, 31.35%, 38.22%, 49.45%, and 49.33%, respectively. Also, the HE staining showed that compared with other groups, the carotid artery thrombosis areas of urokinase group and RGD/ICG/PFP@HMSN group was significantly reduced (Fig. 4H, I). A reduction in the embolic area at the target vessel of the rat was observed following US and NIR treatment (Fig. 4J). Through the assessment and analysis using these methods, combined with monitoring via US/NIR dual-modal imaging, the results are adequate to support the comprehensive determination of vascular recanalization success achieved through the designed research methods. Also, the thrombolytic method was ineffective without either US or NIR irradiation, as well as in the absence of the nanoprobe. Notably, the thrombolytic effectiveness of the urokinase group was similar to that of the RGD/ICG/PFP@HMSN + US + NIR group, underscoring the comparability of both approaches, while the dual-mode thrombolytic strategy in our study is free from non-targeted bleeding risk.

Under the influence of US and NIR, the nanoprobe was activated, leading to thrombolysis. Crucially, the use of both US and NIR has substantiated the synergistic benefits of nanoprobe infiltration in vivo. After the activation of the nanoprobe, microbubbles generated through phase-transition serve as exogenous cavitation nuclei. Under US irradiation, the cavitation threshold of the microbubbles decreases, leading to cavitation effects. Additionally, under NIR exposure, the photothermal efficiency of ICG further elevates the cavitation nucleus temperature of the nanoprobe, synergistically enhancing microbubble rupture. This generates highly energetic transient cavitation, inducing powerful and local physical effects such as shear forces and microjets. Consequently, the surface of the thrombus undergoes a "pinhole" change, exposing the active sites of fibrinolytic enzymes. This exposure promotes the binding of enzymes to fibrin in blood, enhances fibrinolytic enzyme activity, and further accelerates the dissolution of the thrombus. Meanwhile, the thermal effect produced by ICG under NIR has a mild temperature-elevating impact within the blood vessels, effectively loosening fibrin-containing thrombi and also contributing to the synergistic thrombolysis effect. Therefore, this integrated approach, harnessing both sonothrombolysis and photothermal thrombolysis, has resulted in a notable improvement in thrombolysis effectiveness. Our research underscores the potential clinical utility and translational feasibility of this method, which could be one of the viable treatment options. Particularly in scenarios where conventional treatments are unsuitable, it offers an alternative therapeutic strategy or adjunctive therapy to augment treatment efficacy when the therapeutic efficiency of conventional treatments are not satisfied.

### Results of therapeutic safety study

**Fig. 5:**
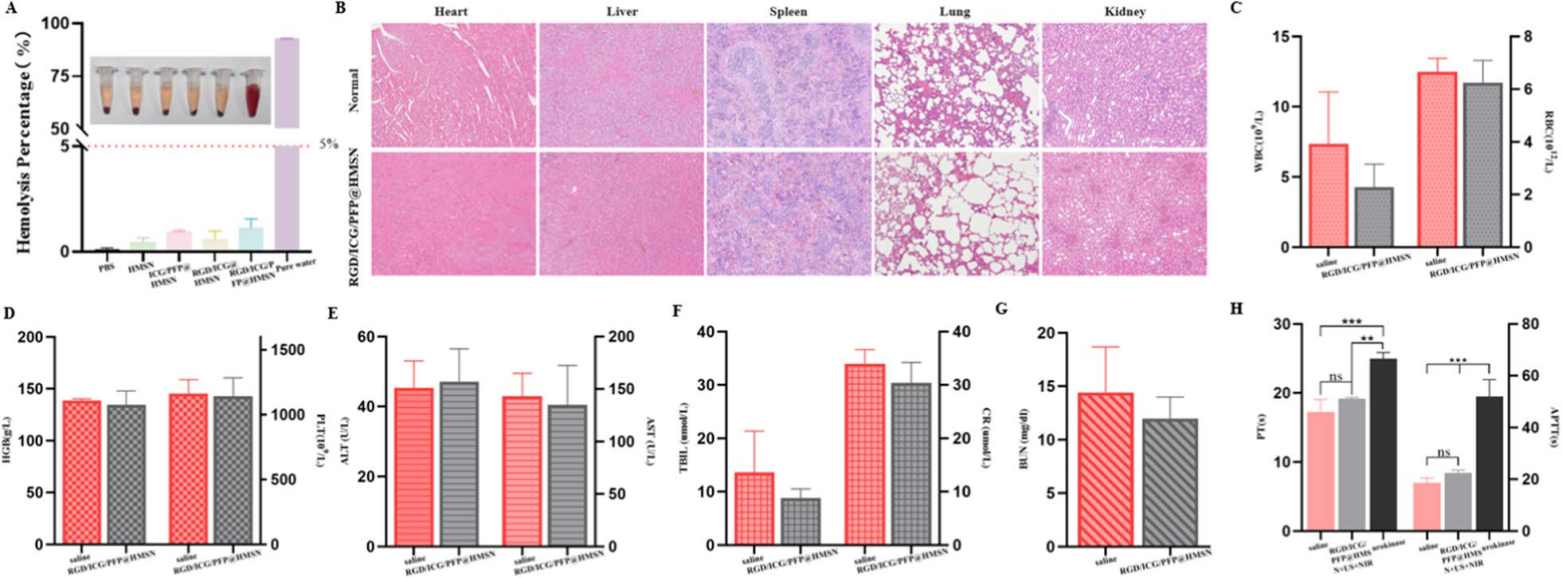
Biosafety study. **A** The Hemolysis experiment. **B** HE staining of the heart, liver, spleen, lung, and kidney of SD rats. **C** WBC and RBC tests of rats, n = 3. (WBC: white blood cell, RBC: white blood cell). **D** HGB and PLT tests of rats, n = 3. (HGB: hemoglobin, PLT: platelet). **E** ALT and AST tests of rats, n = 3. (ALT: alanine aminotransferase, AST: aspartate aminotransferase). **F** TBIL and CR tests of rats, n = 3. (TBIL: total bilirubin, CR: creatinine). **G** BUN test of rats, n = 3. (BUN: blood urea nitrogen). **H** The coagulation indicators of PT and APTT, n = 3. (PT: prothrombin time, APTT: activated partial thromboplastin time)

The hemolysis experiment showed that the hemolysis rate of the negative control group (PBS group) and each experimental group remained well below the upper limit (5%), while the positive control group (pure water group) displayed evident hemolysis (Fig. 5A), which shows satisfied blood compatibility.

After the injection of the nanoprobe, thorough pathological examinations of heart, liver, spleen, lungs, and kidneys of the rats revealed no significant abnormalities (Fig. 5B). Furthermore, routine blood tests (Fig. 5C, D) and function assessments of liver and kidney (Fig. 5E–G) showed no noteworthy irregularities. These findings collectively demonstrated that the nanoprobes were safe for SD rats. Additionally, in contrast to the saline group, the RGD/ICG/PFP@HMSN + US + NIR group exhibited no significant deviations in PT (prothrombin time, PT) and APTT (activated partial thromboplastin time, APTT) examination of the coagulation function, while the coagulation function test of the urokinase group showed that the PT and APTT were significantly prolonged (P < 0.01) (Fig. 5H). These observations indicated that the urokinase group carried a higher risk of bleeding than the RGD/ICG/PFP@HMSN + US + NIR group.

## Conclusion

RGD/ICG/PFP@HMSN designed in our study has demonstrated integrated characteristics specific to thrombus under the dual-mode of US/NIR, with targeting diagnosis and synergistic efficient treatment for both in vitro and in vivo experiments. The innovative strategy of diagnosis and treatment for thrombus paves the way for novel avenues in thrombolysis research and potential clinical applications.

## Experimental section

### Preparation of the RGD/ICG/PFP@HMSN nanoprobe

The synthesis of the RGD/ICG/PFP@HMSN nanoprobe was carried out in a stepwise manner. Initially, ethanol (Sinopharm Chemical Reagent Co., Ltd, Shanghai, China), water, and ammonia (Sinopharm Chemical Reagent Co., Ltd, Shanghai, China) were combined in a round bottom flask and mixed thoroughly at 30 °C with magnetic stirring. Tetraethyl orthosilicate (TEOS, Aladdin Corporation, Shanghai, China) was subsequently introduced into the solution and stirred overnight. A solution of cetyltrimethyllammonium bromide (CTAB, Aladdin Corporation, Shanghai, China) was added to the mixture and stirred to ensure even distribution. The resulting solution underwent multiple centrifugation and cleaning steps. The solution was then redispersed in deionized water and placed in an oil bath at 90 °C for etching over a 24-h period. Afterward, the solution was subjected to further rounds of centrifugation and cleaning and subsequently calcined at high temperature for 6 h following vacuum drying to obtain HMSN.

To prepare RGD/ICG/PFH@HMSN, referring to the methods in the literature [27], specifically, 2 mg of HMSN was transferred into a pre-vacuumed bottle, and 200 μL of PFP (Aladdin Corporation Shanghai, China) was injected into it. The bottle was then subjected to ultrasonic oscillation for 2 min while being immersed in an ice bath [27]. Subsequently, ICG (GlpBio, USA) was introduced to the mixture and stirred in the dark at 4 °C. After 8 h, the mixture was centrifuged and washed. The resulting precipitate was resuspended in 250 μL of MES buffer (Yuanye Biotechnology Co. Ltd. Shanghai, China), and 200 μL of RGD peptide (Jier Biochemical Co. Ltd. Shanghai, China) was added. The mixture was stirred evenly at 4 °C for 30 min. Next, 200 μL of 1-Ethyl-3-(3-dimethyllaminopropyl) carbodiimide hydrochloride (EDC, Aladdin Corporation Shanghai, China) was introduced, and the stirring continued overnight at 4 °C. The mixture then underwent additional rounds of centrifugation and washing, resulting in the green product, designated as RGD/ICG/PFP@HMSN.

### Characterization of the RGD/ICG/PFP@HMSN

The microstructure of nanoprobe was assessed using transmission electron microscopy (TEM, Hitachi TEM system, Hitachi, Japan). To determine the particle size of the nanoprobe, a particle size potentiometer (Zetasizer Nano ZSP, Malvern, UK) was employed. The coupling rate of RGD was quantified using a BCA protein concentration assay kit (Biyuntian Biotechnology Co. Ltd. Shanghai, China). UV visible near-infrared spectroscopy of the nanoprobe was acquired by using ultramicro spectrophotometers (NanoDrop one, Thermo, USA). The standard curve of ICG was generated using Microplate reader (EnSight, Perkin Elmer, USA), and the loading efficiency of ICG was calculated based on the standard curve. To confirm the successful loading of PFP, samples from the PFP group, HMSN group, HMSN + PFP group (physical mixing of HMSN and PFP), and HMSN containing PFP group were subjected to incubation in a 40 °C water bath and observed for thermal induced phase transition before and after incubation.

### Dual-mode imaging capability of the RGD/ICG/PFP@HMSN in vitro

For NIR imaging, the nanoprobe was exposed to NIR live imaging system (series II 900/1700, Suzhou Yingrui Optical Technology Co., Ltd, Jiangsu, China) at different concentrations (0.01 mg/mL, 0.02 mg/mL, 0.05 mg/mL, 0.1 mg/mL, 0.2 mg/mL, 0.5 mg/mL, 1.0 mg/mL, 2.0 mg/mL). Changes in fluorescence intensity were then recorded by capturing images.

For US imaging, an appropriate amount of RGD/ICG@HMSN and RGD/ICG/PFP@HMSN were placed in 3% agarose gel model. Images were acquired in both gray scale and contrast mode using a US diagnostic and imaging system (LOGIQ E9, General Electric Company, USA) before and after LIFU (LM.SC051 ACA, Institute of Ultrasound Imaging of Chongqing Medical Sciences, China) irradiation.

### Photothermal conversion and thermotropic phase transition capabilities of the RGD/ICG/PFP@HMSN in vitro

The nanoprobe was divided into 5 groups based on different concentrations (0.1 mg/mL, 0.2 mg/mL, 0.5 mg/mL, 1.0 mg/mL, 2.0 mg/mL), with pure water serving as the blank control group. Subsequently, they were exposed to 808 nm laser irradiation (VCL-808nmM0-2W, Beijing Honglan Optoelectronics Technology, China) at the power density of 0.6 W/cm^2^ for 3 min. Additionally, the nanoprobe at the concentration of 0.5 mg/mL was also subjected to 808 nm laser irradiation at three different power densities (0.3 W/cm^2^, 0.6 W/cm^2^, 0.9 W/cm^2^) for 3 min each. Temperature changes in each group were measured using a thermal imager (HM-TPH21-3AXF, Hikmicro, Hangzhou Microimage Software Co., Ltd. China) before and after irradiation.

The thermal-induced phase transition before and after 808 nm laser irradiation (0.6 W/cm^2^) of RGD/ICG@HMSN and RGD/ICG/PFP@HMSN was also observed.

### Thrombus targeted binding and thrombolysis of the RGD/ICG/PFP@HMSN in vitro

Under anesthesia, 1.5 mL of abdominal aorta blood was collected from Sprague Dawley (SD) rats and incubated at 37 °C. Once the blood clot formed, it was divided into small strip-shaped pieces measuring 1 cm × 1 cm × 0.5 cm. These strips were then incubated with the RGD/ICG/PFP@HMSN and ICG/PFP@HMSN for 30 min, followed by repeated rinsing with PBS. After fixation with 4% paraformaldehyde, the samples were embedded in OCT (Optimal Cutting Temperature, OCT) compound and frozen into sections at − 20 °C. To observe the establishment of the nanoprobe in targeting thrombus, an upright fluorescence microscope (BX53, OLYMPUS, Japan) was used.

Blood samples were collected from the abdominal aorta of SD rats under anesthesia and the obtained blood was centrifuged at room temperature (1300 rpm, 10 min). After centrifugation, the supernatant was platelet-rich plasma, and the lower layer material was red blood cells. Sucked the supernatant and red blood cell suspension, mixed them in a 9:1 ratio, added coagulant (1:9), and incubated them in a 37 °C incubator to prepare thrombus. After incubation, weighed the thrombus weight. Each thrombus weighed approximately 100–120 mg, and was washed three times with normal saline, then the excess water was absorbed using filter paper. The experimental groups were designated as follows: RGD/ICG/PFP@HMSN + US + NIR group, RGD/ICG/PFP@HMSN + US group, RGD/ICG/PFP@HMSN + NIR group, HMSN + US + NIR group, Saline + US + NIR group. Each group of blood clots was incubated with the respective solutions and subjected to corresponding US and NIR treatments. Afterward, the clots were washed three times with normal saline. Also, the excess water was absorbed using filter paper, and the weight of the blood clots was recorded before and after treatment. Furthermore, hematoxylin and eosin (HE) staining was performed on the treated blood clots.

### Dual-mode imaging capability of the thrombus-targeting in vivo

The animals were provided by the Animal Experiment Center, Renmin Hospital of Wuhan University, and the animal experiments were approved by the Animal Ethics Committee, Renmin Hospital of Wuhan University (No. 20220805A).

SD rats weighing 180–220 g were anesthetized using isoflurane gas. The fur on the neck was carefully removed, and the rats were securely positioned on the operating table with a rope. To expose the carotid artery, a midline incision was made between the chin and sternum, and fine tweezers were utilized to separate the peripheral muscles. Subsequently, a filter paper saturated with 10% FeCl_3_ was applied to the left carotid artery for 10 min until thrombosis formation. Finally, the residual FeCl_3_ was washed for three times using saline.

For NIR imaging, arterial thrombosis rat models were established using the FeCl_3_-induced method. Rats were intravenously injected with RGD/ICG/PFP@HMSN and ICG/PFP@HMSN at an equivalent concentration of 10 mg/kg. Subsequently, the fluorescence intensity at the target vessel was observed and recorded at different time points.

For US imaging, similarly, FeCl_3_-induced arterial thrombosis rat models were established using the same method. RGD/ICG/PFP@HMSN, RGD/ICG@HMSN were also intravenously injected into the rats. After the arterial thrombosis was irradiated by LIFU, the images of gray scale and contrast mode were acquired using US diagnostic and imaging system (LOGIQ E9, General Electric Company, USA).

### Photothermal conversion efficiency and thrombolysis effect in vivo

In the experiment of detecting the photothermal conversion ability of carotid artery thrombosis in rats, the rats weighing 180–220 g were assigned to four groups: normal group, control group (thrombosis group), ICG/PFP@HMSN group, and RGD/ICG/PFP@HMSN group. Arterial thrombosis rat models were established using the FeCl_3_-induced method, as previously described. Each group was irradiated with laser for 15 min and temperature changes were captured using a thermal imager at different time points.

In the thrombolysis experiment, SD rats weighing 180–220 g were randomly assigned to seven groups: control group, RGD/ICG/PFP@HMSN group, US + NIR group, RGD/ICG/PFP@HMSN + NIR group, RGD/ICG/PFP@HMSN + US group, and urokinase group, RGD/ICG/PFP@HMSN + US + NIR group (n = 3 each). Arterial thrombosis rat models were established using the FeCl_3_-induced method, as previously described. Following corresponding treatments given to each group, the rats were sacrificed. Their embolismic vessels were harvested, washed with normal saline, fixed in 4% paraformaldehyde, and subjected to HE staining to evaluate the effectiveness of the thrombus treatment.

### Therapeutic safety study

Blood samples were collected from the abdominal aorta of SD rats under anesthesia. The samples were then centrifuged at 3000 r/min for 15 min at 4 °C to obtain red blood cells. After washing the red blood cell precipitate three times with PBS buffer, it was diluted 10 times. Subsequently, 0.5 mL of red blood cells was mixed with 0.5 mL of the HMSN, ICG/PFP@HMSN, RGD/ICG@HMSN, RGD/ICG/PFP@HMSN at the same concentrations (0.1 mg/mL) as the experimental groups. As positive control group, 0.5 mL of pure water was mixed with 0.5 mL of red blood cells, and 0.5 mL of PBS buffer was mixed with 0.5 mL of red blood cells to serve as the negative control group. After incubating each group at 37 °C for 4 h, they were centrifuged again (3000r/min, 15 min, 4 °C), and the absorbance of the supernatant was measured at 540 nm using the Microplate reader. The hemolysis rate (%) was calculated using the formula: Hemolysis rate (%) = (A_experimental group_ − A_negative control group_)/(A_positive control group_ − A_negative control group_) × 100%, representing the percentage of hemolysis.

One day after the injection of the nanoprobe into SD rats, blood was collected from the SD rats at blood routine test, hepatic and renal function measurements (n = 3). Furthermore, the main organs, including the heart, liver, spleen, lung, and kidney, were washed with normal saline, fixed in the 4% paraformaldehyde, and subjected to HE staining to evaluate any potential pathological changes.

Additionally, plasma samples were collected from the SD rats in the urokinase group and RGD/ICG/PFP@HMSN + US + NIR group for coagulation function test.

### Statistical analysis

Statistical analysis was performed using Graphpad prism 8 (Graphpad software, USA). Data with normal distribution was expressed as mean ± standard deviation. Independent sample t-test was employed to compare two groups, and one-way ANOVA was used for comparison between multiple groups, with statistical significance indicated by a two-tailed p-value of less than 0.05.

## Data Availability

All data are available in the main text and are available from the corresponding authors upon reasonable request.
